# Hyaline vascular- type Castleman's disease in the hilum of liver: a case report

**DOI:** 10.1186/1757-1626-3-74

**Published:** 2010-03-01

**Authors:** Hossein Karami, Alireza Alam Sahebpour, Maryam Ghasemi, Hasan Karami, Mojdeh Dabirian, Kurosh Vahidshahi, Farzad Masiha, Soheila Shahmohammadi

**Affiliations:** 1 Department of Pediatric Oncology & Hematology, Thalassemia Research Center, Mazandaran University of Medical Sciences, Booali Sina Hospital, Pasdaran Boulevard, Sari, Po Box: 48158-38477, Iran; 2 Department of Pediatric Surgery, Mazandaran University of Medical Sciences, Booali Sina Hospital, Pasdaran Boulevard, Sari, Po Box: 48158-38477, Iran; 3 Department of Pathology, Mazandaran University of Medical Sciences, Booali Sina Hospital, Pasdaran Boulevard, Sari, Po Box: 48158-38477, Iran; 4 Department of Pediatric Gastroenterology, Mazandaran University of Medical Sciences, Booali Sina Hospital, Pasdaran Boulevard, Sari, Po Box: 48158-38477, Iran; 5 Department of Cardiology, Mazandaran University of Medical Sciences, Faculty of Medicine, Sari, PO Box: 48471-91971, Iran; 6 Department of Pediatrics, Mazandaran University of Medical Sciences, Faculty of Medicine, Sari, PO Box: 48471-91971, Iran; 7 Clinical Research Development Center, Booali Sina Hospital, Pasdaran Boulevard, Sari, Po Box: 48158-38477, Iran

## Abstract

**Background:**

Castleman's disease or angiofollicular lymphoid hyperplasia is a rare benign lymph node hyperplasia usually presenting as an asymptomatic mediastinal mass in children. The disease can present at any extra thoracic site with lymphoid tissue such as retroperitoneal, mesentery, axilla, and pelvis. Hepatic localization castleman disease is very rare in children. Herein, we reported a case of Castleman's disease arising from the lymph node in hilum of liver.

**Case presentation:**

A 5 -year-old girl with chief complaint of abdominal pain for two months which exaggerated in last three days was referred to the hospital. On routine physical examination, only a generalized abdominal pain was noticed. Routine laboratory investigations and Chest X-Ray were normal. Abdominal Sonography revealed a 3.7 × 3.1 cm solid mass in the hilum of the liver. On the MRI images, a lobulated mass in the portal hepatic associated with mass effect on the portal vein was visible. Histological examination revealed expansion of mantle zone in lymphatic nodules accompanied by burnt out germinal centers. This pattern was matched with the diagnosis of the hyaline-vascular type of Castleman disease. The patient underwent a laparotomy. The patient had an uneventful postoperative course.

**Conclusion:**

This pattern was matched with the diagnosis of the hyaline-vascular type of Castleman disease.

## Introduction

Castleman's disease, first described in 1956, is a rare lymphoproliferative disorder, which commonly found in mediastinum and lung hila. Extrathoracic site of disease is uncommon but not unknown. Clinically it is also divided in two types: a localized form, which is usually asymtomatic and presented as a mass or swelling, and a multicentric type characterized by fever with chills, anaemia, generalized lymphadenopathy and hepatosplenomegaly [1]. Histologically, the disease is also classified into two separate subtypes: the hyaline vascular and plasma cell variants, the earlier being more common and with greater vascularity. The prognosis of Localized Castleman's diseases is excellent with surgical resection [2].

## Case Report

A 5 -year-old girl with chief complaint of abdominal pain for two months which exaggerated in last three days was referred to the hospital. On routine physical examination, only a generalized abdominal pain was noticed, and the family only complained of nocturia during the period of the disease. Routine laboratory investigations were normal. Chest X-Ray and ESR were within normal range too. The birth weight of the patient was 2500 gr after normal vaginal delivery, and she had normal weight gain.

Abdominal sonography revealed a 3.7 × 3.1 cm solid mass in the hilum of the liver that shifted the kidney to the right.

**Figure 1:**
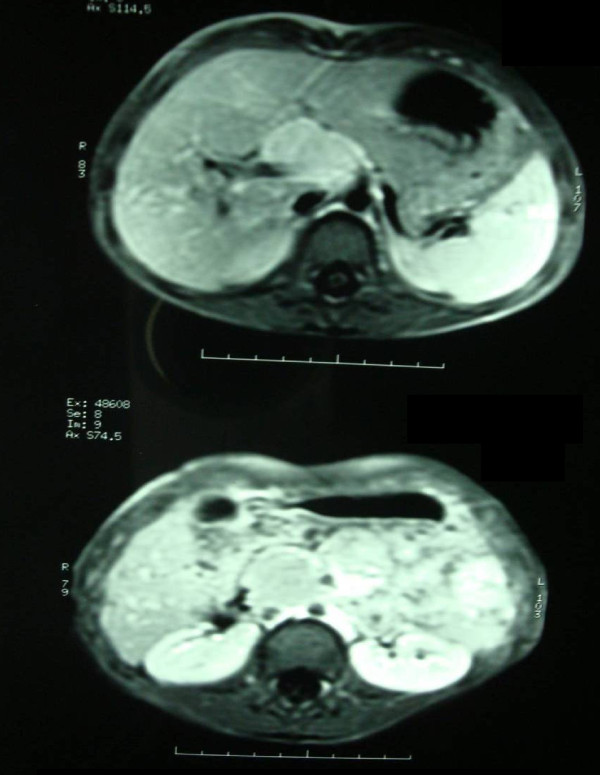
**The T1W images show multiple mass Lesions in hepatic hilum with marked enhancing**.

**Figure 2:**
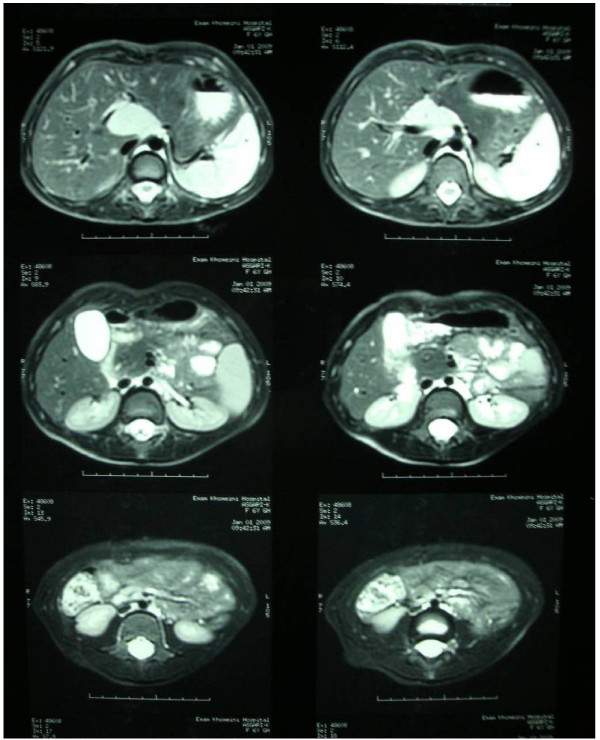
**The T2W images show high signal mass lesions at hepatic hilum**.

**Figure 3:**
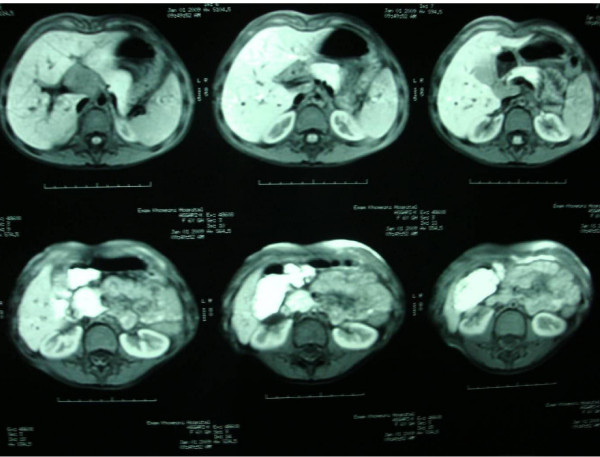
**The post Gd T1W images show enhancing mass lesions at hepatic hilum**.

On the MRI images, a lobulated mass in the portal hepatic associated with mass effect on the portal vein was visible (fig. 1, 2, 3). The mass was low signal intensity on T1 and high signal intensity on T2 weighted images. This was similar to a lymph node. After contrast medium administration, there was enhancement in the mass. The spleen was in normal size and had homogenous internal structure. The pancreas was normal in size and position, and both kidneys were normal in size and position too.

The patient underwent a laparotomy that revealed a solid, homogenous mass adjacent to the right lobe of liver.

**Figure 4:**
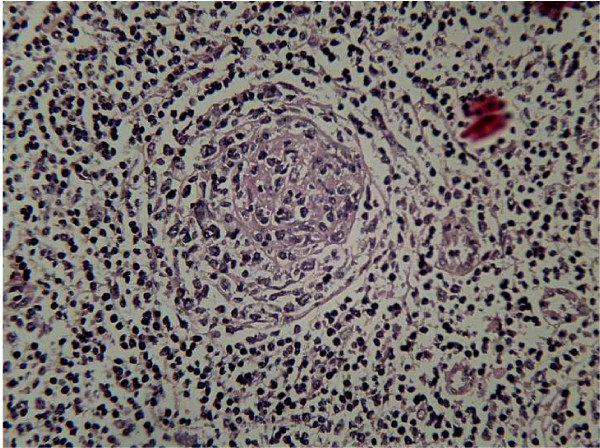
**Hematoxylin and Eosin Stained slide (× 100 magnification)**.

Histological examination revealed essentially preserved lymph node architecture. There was expansion of mantle zone in lymphatic nodules accompanied by burnt out germinal centers. Para cortical areas showed endothelial hyperplasia of vascular channels, some of which encroached the germinal centers (Fig. 4). Immunohistochemical staining was done in which CD34 hard indicated the endothelial hyperplasia of vascular channels, Bcl2 showed marked expansion of the mantle zone. CD23 was negative.

## Discussion

Castleman's disease is a benign and rare vascular lymphoproliferative disorder, which was first reported by Castleman [3] in 1956 while he described a group of patients with large thymomalike masses in the anterior mediastinum.

The etiology of the disease is unknown, characterized by enlargement of hilar and mediastinal lymph nodes. Although the most commonly site of the disease is within the thorax, rare extrathoracic presentations have been described within the abdomen [2], including the left lobe of the liver [4]

Kujat et al. in 1990 described one patient with a lesion arising in the spleen, that described by [5], in which histological examination showed a mixture of both hyaline-vascular and plasma cell types. Levo et al. [6] described a case of multicentric Castleman's disease (MCD) with lesions in the spleen and in lymph nodes throughout the body. In our case, the lesion found in the right lobe of the liver too.

The pathogenesis of Castleman disease is unknown, although most believe that a defect in immunoregulation resulting in excessive proliferation of B lymphocytes and plasma cells in lymphoid organs is responsible for its origin [7].

There are two major pathologic variations. Hyaline-vascular type, the most frequent, characterized by small hyaline-vascular follicles and capillary proliferation; and the plasma-cell type, in which large lymphoid follicles are separated by sheets of plasma cells. The hyaline-vascular cases usually are largely asymptomatic, whereas the less common plasma-cell variant may present with fever, anemia, weight loss, and night sweats, along with polyclonal hypergammaglobulinemia.

The plasma cell variant of Castleman disease is less common and clinically more aggressive [2], with involvement of multiple enlarged lymph nodes. Patients with the localized plasma-cell variant can have systemic manifestations including fever, excessive sweating, fatigue, arthralgia, anemia, elevated erythrocyte sedimentation rate, polyclonal hypergammaglobulinemia, and bone marrow plasmacytosis. These systemic manifestations usually resolve after surgical excision of the mass. Radiographically, these lesions are less vascular and thus demonstrate only mild-to moderate contrast enhancement on CT.

Clinically, Castleman disease also is classified into two groups: localized or disseminated. The previous is associated with a benign outcome; but the disseminated form, on the contrary, is associated with various symptoms, such as fever, splenomegaly, and leukocytosis [8].

Castleman's disease can develop anywhere lymphoid tissue is found. So that, the preoperative diagnosis is often difficult [9]. In our case, diagnosis was made after excision biopsy. Ultrasonography, CT scan, and magnetic resonance imaging have proven to be useful in the diagnosis of disease. However, the images of Castleman disease are similar to other masses including lymphoma, tuberculosis, sarcoidosis, and retroperitoneal sarcomas [10].

Ultrasonography showed a hypoechoic mass in the case reported by Kujat et al., but an isoechoic mass in the case of Levo et al. We found a 3.7 × 3.1 cm solid mass in the hilum of the liver that shifted the kidney to the right.

Castleman's disease typically appears on MRI as low-to-intermediate signals compared with muscle on T1-weighted images and high signals on T2-weighted images.

In review of literature, the T1-weighted images showed masses with low intensity in 13 cases and isointensity in one case (one case was excluded from this study), and the T2-weighted images showed masses with high intensity in 11 cases, high intensity with areas of low intensity in two cases, central low intensity with peripheral high intensity in one case, and iso intensity with areas of high intensity in one case [10]. On our patient's the mass was low signal intensity on T1 and high signal intensity on T2 weighted images.

Patients with the hyaline-vascular variant are usually asymptomatic (97%); however, constitutional symptoms can occur, most often due to the tracheobronchial compression [3]. These symptoms include dyspnea, cough, chest pain, and hemoptysis. This variant is usually benign and self-limited, with a 5-year survival of approximately 100%. However, rare cases have been associated with the development of a Kaposi-type vascular neoplasm [9]. Regardless of the histological type, surgical resection is curative.

## Conclusion

Although the best treatment for Castleman's disease is still unknown, surgical removal of the localized type of the mass has long been considered standard therapy for the disease. Patients with multicentric CD of either histological subtype have a poor prognosis. They are usually treated with a combination of radiation therapy, corticosteroids and chemotherapy. In our patient, a complete surgical excision was accomplished; and up to now, there is no evidence of recurrence.

## Patients' Perspective

According to the parents report, she was suffering from abdominal pain for two months which exaggerated in last three days. After referring to the hospital, on routine physical examination, only a generalized abdominal pain was noticed. She had bed wetting during the period of the disease.

## Abbreviations

MRI: Magnetic resonance imaging.

## Consent

Written informed consent was obtained from the parents of the patient for publication of this case report and accompanying images. A copy of the written consent is available for review by the Editor-in-Chief of this journal.

## Competing interests

The authors declare that they have no competing interests.

## Authors' contributions

HK and FM reporting the case and contributing in writing the manuscript. AAS- medical management of the patient. MG- carried out the pathologic assessment. HK and MD and KV - drafting and revision of the article. SS- contributor in search of articles and references. All authors read and approved the final manuscript.

## References

[B1] Castleman B Towne VW Case records of the Massachusetts General Hospial.: case 40011 N Engl J Med 1954 250 26 30 13165944 10.1056/NEJM195406102502308

[B2] Keller A Hochholzer L Castleman B Hyaline-vascular and plasma-cell types of giant lymph node hyperplasia of the mediastinum and other locations Cancer 1972 29 670 83 10.1002/1097-0142(197203)29:3<670::AID-CNCR2820290321>3.0.CO;2-# 4551306

[B3] Castleman B Towne VW Case report of Massachusetts General Hospital; weekly clinicopathological exercises; founded by Richard C. Cabot. Case 40011 N Engl J Med 1954 250 26 3013111435

[B4] Libson E Fields S Strauss S Bloom RA Okon E Galun E Polliack A Widespread Castleman disease: CT and US findings Radiology 1988 166 753 755 3277245 10.1148/radiology.166.3.3277245

[B5] Von Kujal CH Muller-Leisse CH Lorbacher P Setifert R Falk S Stutte HJ Unifokale manifestation einer Castleman- Erkrankung (angiofollikulare impliatische hyperplasia) in der Milz Fortschr Rontgenstr 1990 152 615 7 10.1055/s-2008-1046936 2160704

[B6] Levo Y Behar AJ Blunt I Frish B A benign course of multicentric Castleman's disease with involvement of the spleen and bone marrrow Eur J Haematol 1987 39 471 4 3691764 10.1111/j.1600-0609.1987.tb01459.x

[B7] Shahidi H Myers JL Kvale PA Castleman's disease Mayo Clin Proc 1995 70 969 977 10.4065/70.10.969 7564550

[B8] Herrada J Cabanillas F Rice L Manning J Pugh W The clinical behavior of localized and multicentric Castleman disease Ann Intern Med 1998 128 657 62 9537940 10.7326/0003-4819-128-8-199804150-00010

[B9] Sato Nagato Kondo Satoshi Saito Katsunori Hirano Satoshi Hara Takashi Tanaka Eiichi Ootake Setsuyuki Shichinohe Toshiaki Kawarada You Takeuchi Mikiya Miyamoto Masaki Morikawa Toshiaki Hyaline Vascular-Type Castleman's Disease in the Hepatoduodenal Ligament: Report of a Case Surg Today 2006 36 647 650 10.1007/s00595-006-3200-2 16794803

[B10] Ko SF Hsieh MJ Ng SH Lin JW Wan YL Lee TY Chen WJ Chen MC Imaging spectrum of Castleman's disease Am J Roentgenol 2004 182 769 75 10.2214/ajr.182.3.1820769 14975984

